# Poverty and family adversity trajectories and not in education, employment or training (NEET) status in late adolescence: evidence from the UK Millennium Cohort Study

**DOI:** 10.1136/bmjph-2025-003958

**Published:** 2026-03-30

**Authors:** Kalu Udu, Nicholas Kofi Adjei, Lateef Akanni, Gianmaria Niccodemi, Yanhua Chen, Yu Wei Chua, Rosalie Cattermole, Michelle Black, Luke Munford, Karsten Thielen, Leonie K Elsenburg, Steven Hope, Hanna Creese, Dougal Hargreaves, Naja Hulvej Rod, David Taylor-Robinson

**Affiliations:** 1 Department of Public Health, Policy and Systems, University of Liverpool, Liverpool, UK; 2 Department of Urban Studies and Social Policy, University of Glasgow, Glasgow, Scotland; 3 School of Health Sciences, University of Manchester, Manchester, UK; 4 Department of Occupational and Social Medicine, University Hospital of Holbæk, Holbæk, Denmark; 5 Copenhagen Health Complexity Center, Department of Public Health, University of Copenhagen, Copenhagen, Denmark; 6 School of Public Health, Imperial College London, London, UK

**Keywords:** Education, Public Health, Sociodemographic Factors

## Abstract

**Background:**

Young people who are not in education, employment or training (NEET) are at an increased risk of long-term social and economic disadvantage. While previous research has linked various risk factors and individual characteristics to NEET status, evidence on the cumulative impact of early-life exposure to childhood adversity in the UK remains limited. We therefore aimed to investigate the association between trajectories of poverty and family adversity and NEET status in late adolescence, and to characterise socioeconomic differences, including household income and maternal education by NEET status.

**Methods:**

We analysed longitudinal data on 8368 participants from the UK Millennium Cohort Study. Using a group-based multi-trajectory modelling approach, we identified six distinct exposure trajectories of poverty and family adversity (ie, low poverty and adversity, persistent poverty, persistent poor parental mental health, persistent parental alcohol use, persistent domestic violence and abuse, and persistent poverty and poor parental mental health) from aged 9 months to 14 years. NEET status was assessed at age 17. Adjusted ORs (aORs) and 95% CIs were estimated using logistic regression models. Population attributable fractions were calculated to estimate the proportion of NEET cases attributable to childhood poverty and family adversity.

**Results:**

Overall, 3.5% of participants were NEET at age 17 years. NEET status was more prevalent among young people from socially disadvantaged backgrounds than their peers. Exposure to persistent family childhood adversities was associated with greater likelihood of being NEET. Young people exposed to both persistent poverty and poor parental mental health throughout childhood (9 months to 14 years) had five times greater odds of being NEET (aOR 5.0; 95% CI 3.4 to 7.5) compared with those in low poverty and adversity. An estimated 52.9% (95% CI 41.1% to 61.7%) of NEET cases were attributable to persistent exposure to poverty and family adversity.

**Conclusion:**

Family childhood adversities, particularly household poverty and poor parental mental health, are strongly associated with an increased risk of being NEET on transition to adulthood. Interventions that address early-life socioeconomic disadvantage and family functioning may be critical for preventing NEET and mitigating its long-term social and economic consequences.

**WHAT IS ALREADY KNOWN ON THIS TOPIC**

- Young people who are not in education, employment or training (NEET) are at risk of poor health, social exclusion and long-term economic disadvantage.
- Childhood poverty and family adversity have been associated with NEET status, but their cumulative and life-course impact in the UK remains unclear.

**WHAT THIS STUDY ADDS**

- Using longitudinal data from a nationally representative UK cohort, this study shows that persistent exposure to poverty and family childhood adversity including poor parental mental health increases the likelihood of being NEET at age 17.
- Individuals exposed to multiple family childhood adversity (ie, poverty and poor parental mental health) were five times more likely to be NEET.
- An estimated 52.9% NEET cases were attributable to persistent poverty and family childhood adversity.

**HOW THIS STUDY MIGHT AFFECT RESEARCH, PRACTICE OR POLICY**

- Interventions that address family childhood adversity, particularly household poverty and poor parental mental health, could substantially reduce NEET prevalence and mitigate long-term inequalities.

## Introduction

 Young people who are not in education, employment or training (NEET) are widely recognised as a vulnerable population,^1 2^ with significant implications for social and public health policy. The concept of NEET has gained considerable attention globally and it is increasingly used by policymakers as a key measure of youth disengagement from the labour market.^1 3 4^ In the UK, approximately 13.4% of young people aged 16–24 were classified as NEET by the end of 2024, marking an increase of over 100 000 compared with the previous year.^5^

NEET status has been consistently shown to be associated with a range of adverse outcomes including poor physical health, mental health and early mortality.^6^,^9^ The consequences of non-participation in education, employment or training extend beyond health outcomes.^1^ At the societal level, being NEET poses significant social and economic costs, including lost productivity, increased welfare dependency and employment challenges.^3 10^ Prior evidence also suggests that even brief periods of non-participation from employment, education or training during adolescence may have an enduring ‘scarring’ effect,^11^ diminishing future employment prospects and reducing future earning potential.^12^

Existing literature has identified a range of risk factors for NEET status, including low educational achievement, psychosocial difficulties and substance use.^13^,^20^ More recently, growing attention has focused on the role of early childhood adversities such as poverty, material deprivation, exposure to traumatic events^21^ and parental mental illness.^22^ These adversities can disrupt normative developmental processes and may further erode the emotional, cognitive and social capacities for a successful transition into adulthood.^23^

Recent Danish population-based registry studies have shown how patterns and trajectories of childhood adversity influence a range of adverse adult outcomes, including NEET. Using the DANLIFE cohort, Elsenburg and colleagues identified that exposure to cumulative adversity trajectories was strongly associated with increased risk of being NEET in early adulthood.^22^ In a complementary analysis, Bennetsen *et al*^24^ showed that early school leaving partially mediated the long-term association between childhood adversity and long-term social benefit, highlighting the role of disrupted education pathways. Further evidence using the DANLIFE cohort demonstrated that individuals exposed to adversities had markedly higher use of health, social and justice services in early adulthood,^25^ underscoring the broad and intersecting impacts of childhood disadvantage and adversities on public systems.

These studies offer compelling insights into the complexity and clustering of childhood adversities using rich, linked administrative data. However, to date, no analyses have assessed these relationships in the UK context. Building on our previous study on the clustering of poverty and family adversity across childhood,^26^ and adopting a life-course perspective,^27^ this study aimed to: (1) examine the association between trajectories of poverty and family adversities from infancy to early adolescence (9 months to 14 years) and NEET status at age 17 and (2) assess how structural indicators of disadvantage, including household income and maternal education, are related to NEET risk, thereby situating patterns of childhood adversity within the wider context of socioeconomic inequality. A life-course approach is essential for understanding how long-term and combined exposure to multiple childhood adversities shape developmental pathways and transitions from adolescence into early adulthood.^27^,^29^

## Methods

### Study setting and participants

We used data from the Millennium Cohort Study (MCS), a large-scale longitudinal study tracking over 18 000 children born in the UK (England, Northern Ireland, Scotland and Wales) between September 2000 and January 2002. The cohort has been followed through multiple survey waves, with data collection occurring when the children were approximately 9 months, 3 years, 5 years, 7 years, 11 years, 14 years and 17 years. The age 17 data were collected from January 2018 to April 2019. The numbers of participants at each wave were 18 552, 15 590, 15 246, 13 857, 13 287, 11 726 and 10 625, respectively. Information was collected directly from the main caregiver, usually the child’s mother, and more recently, from cohort members themselves. The MCS used a stratified, clustered sample design, with clustering at the electoral ward level and oversampling of disadvantaged and ethnically diverse areas.^30^ To account for socially patterned non-response and attrition over time, we used longitudinal survey weights that combine sampling design weights with adjustments for differential loss to follow-up. These weights were applied in all descriptive and regression analysis. Further details on response patterns, sampling methodology and survey design are available in the MCS technical documentation.^31^ Each data collection wave has received National Health Service (NHS) research ethics approval, and the analysis reported here did not require additional ethical approval.

### Outcome and exposures

The main outcome for this study was NEET status at age 17. We derived a binary indicator of NEET status using responses from cohort members to four questions: (1) ‘Are you currently going to school or college?’, (2) ‘Are you currently doing an Apprenticeship?’, (3) ‘Are you currently doing any kind of traineeship, training course or scheme?’ and (4) ‘Are you currently doing any kind of paid job?’ Participants who responded ‘no’ to all four items were classified as NEET; those who responded ‘yes’ to at least one were classified as not NEET.

Our primary exposures were relative income poverty and family adversity at different follow-up points from infancy through early adolescence (9 months to 14 years). Poverty was defined as household equivalised income below 60% of the national median, according to the Organisation for Economic Co-operation and Development household equivalence scale.^32^ Family adversity comprised repeated exposure to parental mental health, domestic violence and abuse and alcohol use (see online supplemental table S1 for the list and description of measures). In our previous study, we identified six distinct trajectories of poverty and family adversity,^26^ using group-based multi-trajectory modelling approach^28^: (1) the ‘low poverty and adversity’ group (45.1%) includes children with low overall exposure to childhood poverty and family adversity; (2) the ‘persistent poverty’ group (21.0%) includes children with a high probability of experiencing poverty throughout childhood; (3) the ‘persistent poor parental mental health’ group (12.0%) is mainly characterised by high rates of poor parental mental health over time; (4) the ‘persistent parental alcohol use’ group (8.2%) includes children with a high probability of being exposed to parental alcohol use throughout childhood; (5) the ‘domestic violence and abuse’ group (3.5%) includes children with a high probability of having been exposed to domestic violence throughout childhood and (6) the ‘persistent poverty and poor parental mental health’ group (10.1%) includes children with high exposure to the co-occurrence of persistent poverty and poor parental mental health over time. These trajectory groups were used in this present study as the main exposure variables.^26^

### Confounders

Confounders were selected based on their potential association with the exposure (relative income poverty and family adversity) and the outcome (NEET),^33^ guided by a directed acyclic graph (online supplemental figure S1). We adjusted for maternal education, maternal ethnicity and child sex, all measured when the child was aged 9 months. Maternal education was classified according to the highest academic qualification achieved by the mother (degree or higher, diploma, A-levels, GCSEs A*–C, GCSEs D–G or no formal qualifications). For international comparison, these categories can be interpreted in terms of approximate duration of completed education, reflecting the typical length of schooling in the UK: degree or higher (≥16 years), diploma or A-levels (13–15 years, depending on qualification type), GCSEs (11–13 years) and no formal qualifications (<11 years). Previous analyses have shown maternal education to be closely associated with family income and with children’s educational engagement across the early life course.^34^ Maternal ethnicity was classified using the six-category census-based grouping (white, mixed, Indian, Pakistani and Bangladeshi, black or black British and other ethnic groups),^35^ and was included to account for broader structural and social patterning in exposure to poverty and subsequent educational outcomes.^36^ Child sex (male or female) was included as a baseline covariate.

### Statistical analysis

First, exposure trajectories were characterised from age 9 months to 14 years using group-based multi-trajectory models.^26^ This approach identified subgroups of cohort members who followed similar patterns of adversity from childhood to early adolescence. We then described sample characteristics across the six estimated trajectory groups using percentages (%). In addition, we estimated the prevalence of being NEET at age 17 by maternal education and household income quintile. To assess the associations between the trajectory groups and NEET status, we fitted logistic regression models and reported ORs with 95% CIs, adjusting for potential confounders. The model incorporated longitudinal survey weights to account for non-response, attrition and sampling design. To assess the robustness of the adjusted association to potential unmeasured confounding, we calculated E-values for the main estimates.^37^ The E-values represent the minimum strength of association that an unmeasured confounder would need to have with both the exposure and the outcome, beyond measured covariates, to fully explain away the observed association. Finally, we estimated population attributable fractions (PAFs) to quantify the proportion of NEET cases that could be potentially prevented if exposure to poverty and family adversity were reduced to the levels observed in the low poverty and adversity trajectory group. PAFs and their corresponding 95% CIs were calculated using the ‘punaf’ command in STATA (see technical description S1 for more details on the model specification). All analyses were conducted using STATA V.16.0.

## Results

### Study population characteristics

Of the 10 625 participants at age 17 (wave 7), 8368 were included in the final analysis (online supplemental figure S2). Table 1 presents baseline sociodemographic characteristics of cohort members across the six trajectory groups. Estimates derived using multiple imputation by chained equation (n=25) are provided in online supplemental table S2. Marked differences were observed across trajectories for maternal ethnicity and maternal education. For instance, membership in the trajectory characterised by persistent poverty and poor parental mental health was more common among children whose mothers had no formal educational qualifications.

**Table 1:** Baseline characteristics by the six estimated trajectory groups, observed data

Characteristics	Family adversity and poverty trajectories						Overall (n=8368)
	Low poverty and adversity (n=3846)	Persistent parental alcohol use (n=706)	Persistent domestic violence and abuse (n=300)	Persistent poor parental mental health (n=991)	Persistent poverty (n=1714)	Persistent poverty and poor parental mental health (n=811)	
Child’s sex							
Boy	1815 (47.2%)	319 (45.2%)	152 (50.7%)	463 (46.7%)	707 (41.2%)	392 (48.3%)	3848 (45.9%)
Girl	1941 (50.5%)	361 (51.1%)	144 (48.0%)	498 (50.3%)	923 (53.9%)	363 (44.8%)	4230 (50.6%)
Missing	90 (2.3%)	26 (3.7%)	4 (1.3%)	30 (3.0%)	84 (4.9%)	56 (6.9%)	290 (6.5%)
Maternal education							
Degree plus	1187 (30.7%)	309 (43.8%)	69 (23.0%)	211 (21.3%)	43 (2.5%)	14 (1.7%)	1833 (21.9%)
Diploma	503 (13.1%)	81 (11.5%)	47 (15.7%)	91 (9.2%)	55 (3.2%)	16 (2.0%)	793 (9.5%)
A-levels	499 (12.9%)	69 (9.8%)	40 (13.3%)	116 (11.7%)	106 (6.2%)	35 (4.3%)	865 (10.3%)
GCSE A–C	1162 (30.2%)	161 (22.8%)	88 (29.3%)	350 (35.3%)	514 (30.0%)	233 (28.7%)	2508 (30.0%)
GCSE D–G	192 (5.0%)	25 (3.5%)	27 (9.0%)	89 (8.9%)	232 (13.5%)	114 (14.1%)	679 (8.1%)
None	212 (5.6%)	35 (5.0%)	25 (8.3%)	103 (10.4%)	674 (39.3%)	336 (41.4%)	1385 (16.6%)
Missing	91 (2.5%)	26 (3.6%)	4 (1.4%)	31 (3.2%)	90 (5.3%)	63 (7.8%)	305 (3.6%)
Maternal ethnicity							
White	3442 (89.5%)	665 (94.2%)	252 (84.0%)	821 (82.9%)	1006 (58.7%)	465 (57.3%)	6651 (79.5%)
Mixed	19 (0.5%)	5 (0.7%)	6 (2.0%)	7 (0.7%)	27 (1.6%)	16 (2.0%)	80 (1.0%)
Indian	118 (3.1%)	2 (0.3%)	17 (5.7%)	36 (3.6%)	52 (3.0%)	19 (2.3%)	244 (2.9%)
Pakistani and Bangladeshi	41 (1.1%)	0 (0.0%)	5 (1.7%)	28 (2.8%)	391 (22.8%)	193 (23.8%)	658 (7.9%)
Black or black British	79 (2.1%)	3 (0.4%)	12 (4.0%)	26 (2.6%)	110 (6.4%)	37 (4.6%)	267 (3.2%)
Other ethnic groups	50 (1.3%)	4 (0.6%)	4 (1.3%)	41 (4.1%)	39 (2.3%)	22 (2.7%)	160 (1.9%)
Missing	97 (2.4%)	27 (3.8%)	4 (1.3%)	32 (3.3%)	89 (5.2%)	59 (7.3%)	308 (3.6%)

GCSE, General Certificate of Secondary Education.

**Figure 1:**
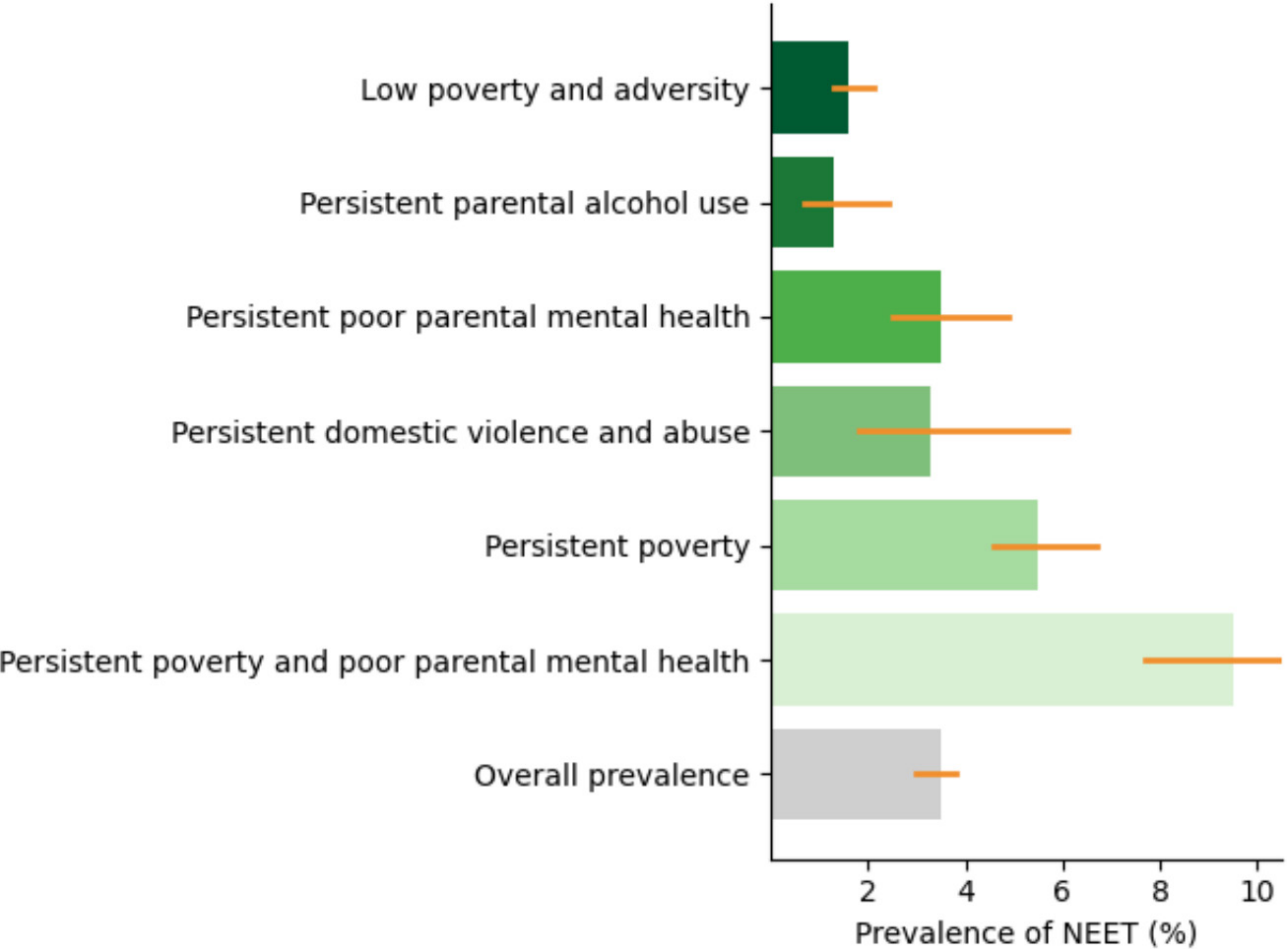
Prevalence (%) and CIs (95% CI) of being NEET in the UK at age 17 by poverty and family adversity trajectories. NEET, not in education, employment or training.

Overall, 3.5% (95% CI 3.0% to 3.8%) of cohort members reported being NEET at age 17. Figure 1 shows the prevalence of NEET by trajectory group. NEET prevalence increased across trajectories characterised by greater exposure to poverty and family adversity. For example, the prevalence of NEET was 9.5% (95% CI 7.7% to 11.7%) among children in the persistent poverty and poor parental mental health trajectory, compared with 1.6% (95% CI 1.3% to 2.1%) among those in the low poverty and adversity trajectory.

**Figure 2:**
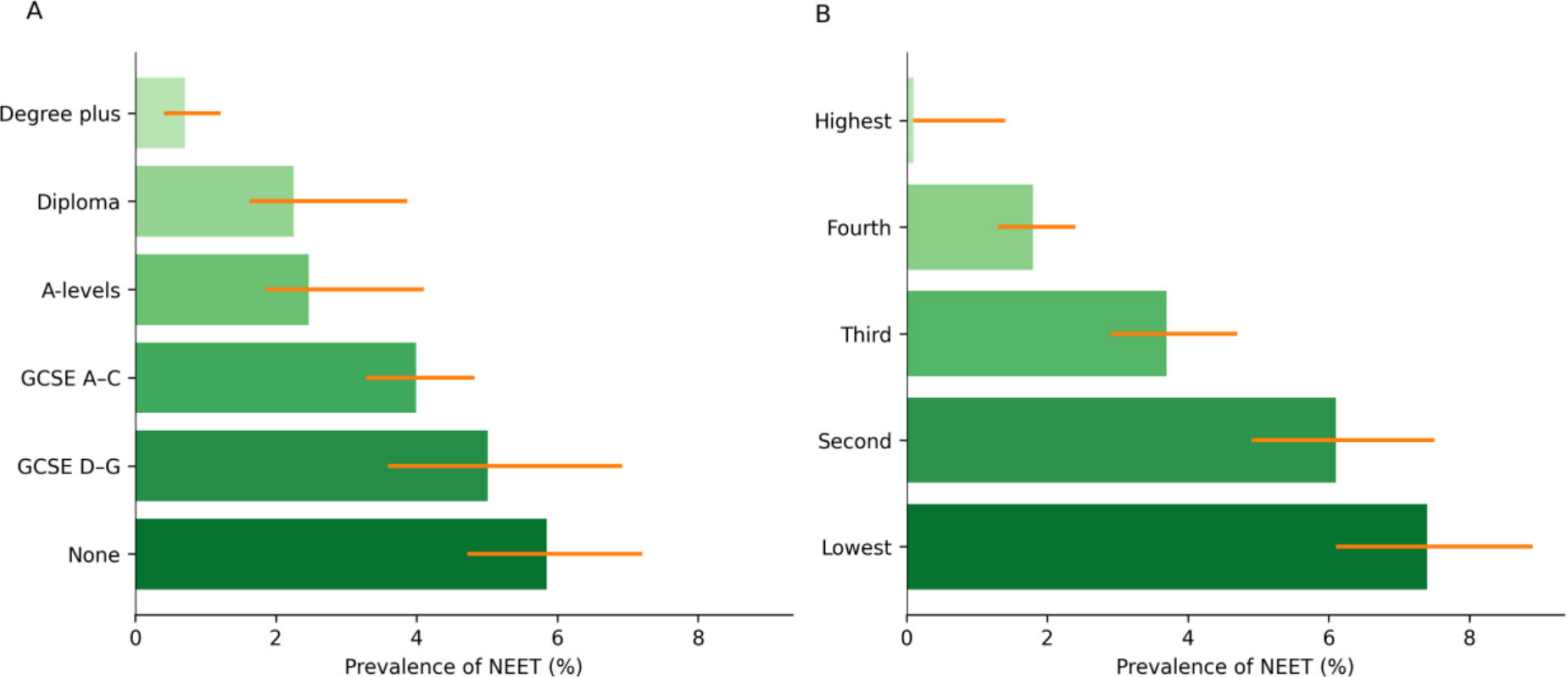
Prevalence (%) and CIs (95% CI) of being NEET in the UK at age 17 by maternal education (panel A) and household income quintile (panel B) at birth. GCSE. General Certificate of Secondary Education; NEET, not in education, employment or training.

Figure 2 shows the prevalence of NEET status by maternal education level and household income quintile. NEET status in early adulthood was more prevalent among young people from socially disadvantaged backgrounds. For instance, those born to mothers with lower educational qualifications were more likely to be NEET (Education—degree plus: 0.7% vs no qualifications: 5.9%). An even steeper social gradient was observed with household income, where NEET prevalence was 7.4% in the lowest income quintile compared with 0.1% in the highest.

### Associations of poverty and family adversity trajectories with NEET status

**Figure 3:**
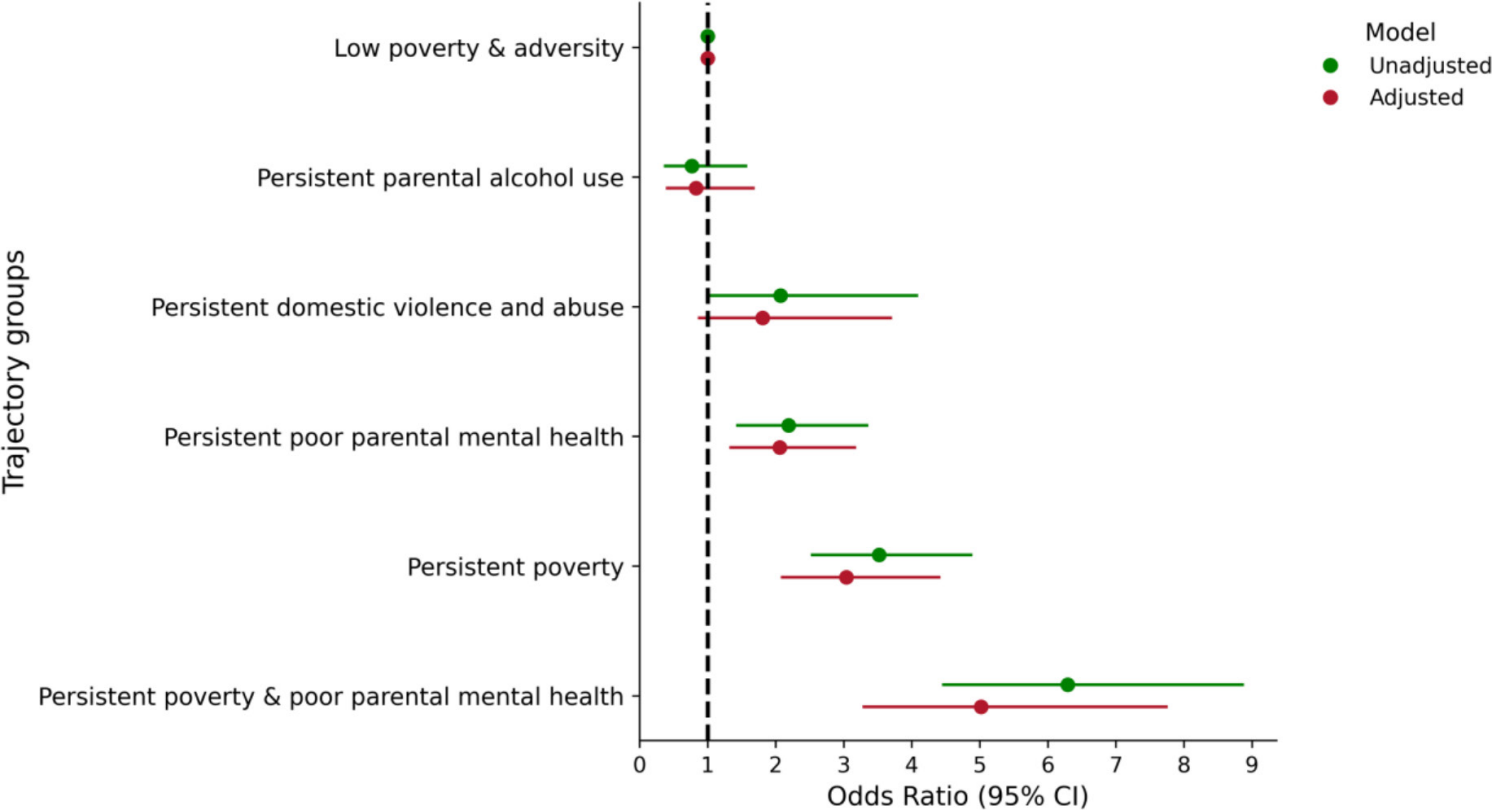
Associations of family adversity and poverty trajectories and being NEET at age 17 years in the UK Millennium Cohort Study. NEET, not in education, employment or training.

Figure 3 shows the associations between adversity trajectory group membership and NEET status at age 17. In both the unadjusted model and the adjusted model, participants in trajectories characterised by persistent poverty and family adversity generally had higher odds of being NEET compared with those in the low poverty and adversity trajectory group. Associations were strongest among those exposed to both persistent poverty and poor parental mental health. For example, the odds of being NEET were five times higher among participants in this trajectory group compared with those in the low poverty and adversity group (adjusted OR 5.0; 95% CI 3.4 to 7.5). The regression analysis was also repeated using imputed data (ie, multiple imputations by chained equations (n=25)) (online supplemental table S3), and the estimates were similar to the main results.

### Population attributable fraction

**Figure 4:**
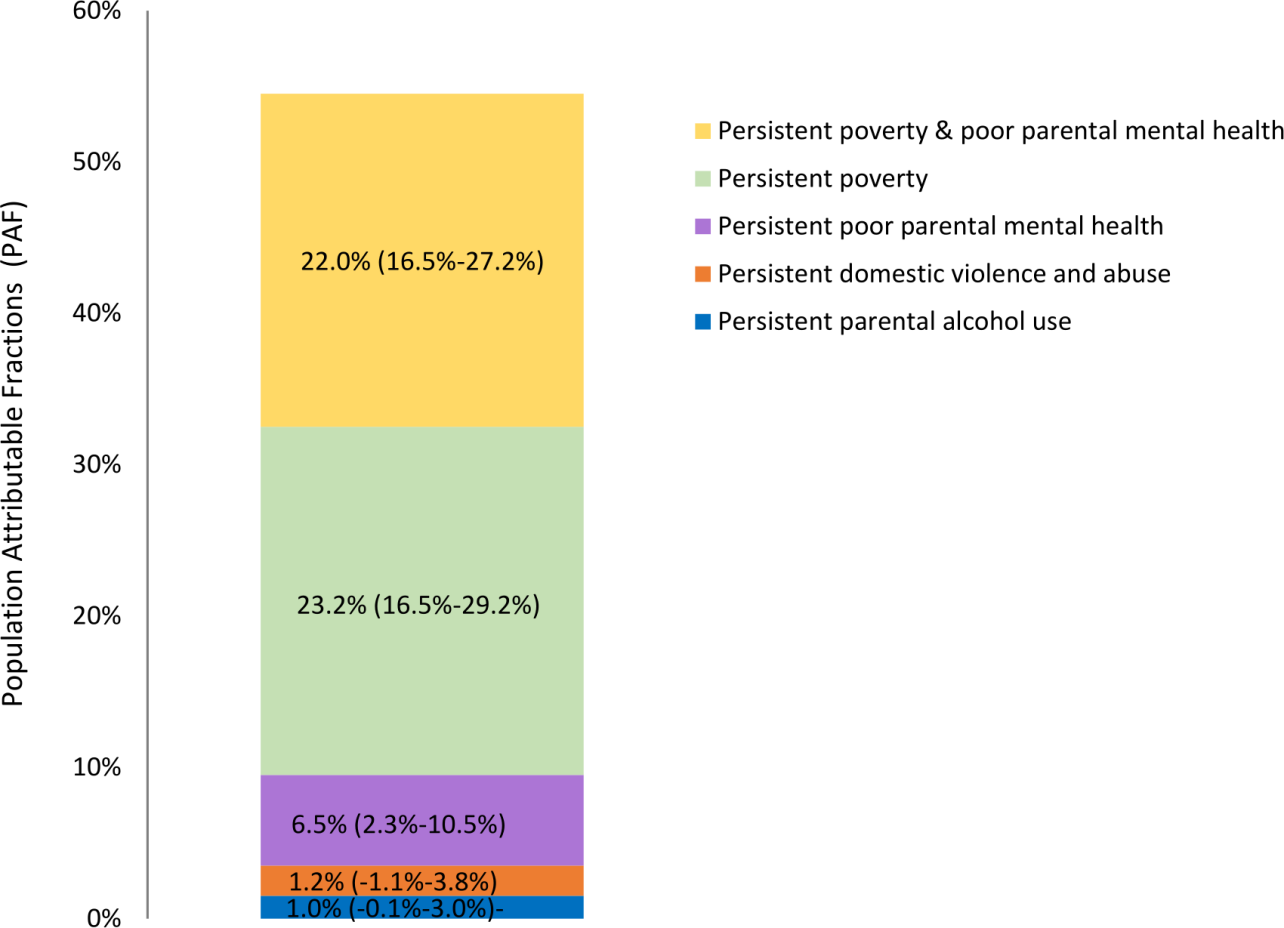
Population attributable fractions of trajectory groups, Compared with the low poverty and family adversity trajectory groups, the overall proportion of being NEET attributable to persistent poverty and family adversity was 52.9% (95% CI 41.1% to 61.7%). Model adjusted for child’s sex, maternal education and ethnicity.

The PAF estimates show the proportion of NEET cases at age 17 that were attributable to each trajectory group (figure 4), assuming a causal relationship. Approximately 53% of NEET cases at age 17 were attributable to persistent poverty and family adversity during childhood. This suggests that, if all children had experienced the trajectory characterised by low poverty and family adversity, the number of NEET cases could be reduced by more than half. When broken down into individual trajectories, exposure to persistent poverty alone and in combination with persistent poor parental mental health accounted for the largest proportion (>50%) of the population-level burden.

## Discussion

In a large contemporary, population-based cohort from the UK, we show that trajectories of poverty and family adversity across childhood are associated with the risk of being NEET at age 17. Persistent exposure to poverty, particularly when combined with poor parental mental health, was associated with markedly higher risks of NEET status. We found that 3.5% of young people aged 17 reported being NEET, consistent with the national estimate of 3.6% for 16–17 years olds in 2017^38^—the year participants in the MCS were also around age 17. More importantly, our findings indicate that over half of NEET cases in this cohort were attributable to childhood poverty and family adversity including poor parental mental health.

Our findings are similar to those in the recent Danish register-based study by Elsenburg and colleagues,^22^ whereby children experiencing high levels of adversity had around five times greater risk of being NEET in two consecutive years.^22^ Our results are also consistent with prior studies identifying socioeconomic disadvantage, parental unemployment and mental health problems as key risk factors for NEET status.^13^,^16^ The Danish studies and this current analysis highlight the importance of life-course analyses, capturing the clustering and cumulative burden of poverty and other family adversities from infancy through to transition to adulthood.^27 28^ Life-course and trajectory-based approaches have been shown to be useful in modelling how early exposures shape long-term outcomes and how the clustering of multiple adversities can have strong negative effects on young people’s life chances.^39 40^

Indeed, the strength of the associations observed, particularly among those exposed to both persistent poverty and poor parental mental health, suggests a heightened vulnerability.^41^ While we did not formally test for interactions over time, the findings are consistent with the possibility that social disadvantage may operate in a cumulative and synergistic manner.^26 40^ The underlying mechanisms are likely to operate through multiple pathways.^42^ For example, parental mental health illness may impact children directly through impaired emotional bonding and increased family conflict^41^ and indirectly by contributing to poor academic performance and social, emotional and behavioural problems.^43 44^ These risk factors may form a critical component of the pathway to later NEET status. In fact, academic attainment, school attendance and engagement are known to be key mediators in the pathway between childhood adversity, labour market disengagement,^1 45^ and these risk factors may further be intensified by reduced access to educational support and structural inequalities.^1 43 45^ The steep socioeconomic gradient in NEET prevalence marked by higher risk among young people from low-income households and those born to less educated mothers, observed in this study, is consistent with broader literature on the intergenerational transmission of disadvantage.^46 47^

A key strength of this study is the use of a large, nationally representative cohort and the application of a robust trajectory modelling approach to capture patterns of childhood adversity over time.^28 29^ Nonetheless, some limitations should be acknowledged. First, the NEET outcome was measured at a single time point and may not reflect temporal patterns or transitions in and out of education or employment.^22^ However, as cohort members were aged 17, this outcome measure reflects a critical period immediately following the end of compulsory education. Second, although we adjusted for relevant confounders, residual confounding from unmeasured factors such as school-level factors and peer effects and disabilities may persist.^48^ Nonetheless, we assessed the robustness of unmeasured confounding using the E-values approach,^37^ and we found that our findings are robust to omitted confounding (online supplemental figure S3). Third, there are also some limitations related to the use of PAFs. For instance, PAFs rest on the assumption that the observed relationship between exposure and outcome reflects a true causal relationship.^49^ However, when interpreted cautiously, PAFs provide a useful tool for estimating the potential population-level impact of modifiable exposures, offering valuable insights for public health planning and policy prioritisation.^49^ Fourth, the exposure is limited to poverty and family adversity measures. We lacked data on other dimensions of childhood adversity such as parental psychiatric illness and unemployment, which has been shown to impact later life.^40^ Fifth, missing data and attrition are common sources of bias, particularly selection bias, in longitudinal studies. Nonetheless, we addressed part of the missing data using multiple imputation. Sixth, NEET status is a pragmatic policy indicator rather than a precise analytic construct and has been criticised for aggregating heterogeneous groups with different risks, resources and needs.^50^,^52^ As a result, a binary measure may obscure important differences in lived experiences and pathways,^51^ particularly between structurally marginalised young people and those who are temporarily or voluntarily disengaged. In our study, NEET status was self-reported at age 17 and therefore likely captured a range of motivations and constraints. We therefore interpret our findings as identifying structural patterning of risk at a key transition point, rather than implying a uniform mechanism or need across all individuals classified as NEET. Finally, our analysis did not directly examine possible mediating factors such as adolescent mental health, academic attainment or school exclusion, which likely lie on the causal pathway. These mechanisms warrant further investigation in future research. Notwithstanding these limitations, NEET as framed in our study is a major focus of UK government policy, and we believe that the results will inform ongoing strategy development.

### Policy implications

Our findings have important implications for both social and public policy. PAF estimates suggest that, assuming a causal relationship, up to half of NEET cases in this cohort could potentially be prevented if exposure to poverty and family adversity throughout childhood were eliminated. From a public health perspective, addressing early-life poverty and family adversity presents a powerful opportunity to reduce NEET and promote social and economic resilience.^53^ Our analysis suggests that this approach could lead to a large reduction in the burden of NEET with huge cost savings across all sectors. For instance, according to the most recent population estimates, there were around 1 567 000 16–17 year olds in the UK in 2023.^54^ The figures estimated here suggest that 3.5% of these, around 54 850 16–17 year olds, will be NEET. Applying cost tariffs from HM Treasury’s Green Book^55^ indicates that the average yearly cost to the government, comprising benefit payments and foregone tax and national insurance contributions, is £11 474 per-NEET-per-year. Analysis in this current study shows that an estimated 52.9% (41.1% to 61.7%) of NEET cases were attributable to persistent exposure to poverty and family adversity. Therefore, if exposure to poverty and family adversity were eliminated, that is, if all children experienced the low poverty and adversity trajectory, NEET prevalence could reduce by approximately 1.85% (95% CI 1.23% to 2.41%) across all 17-year-olds, equivalent to around 29 000 (95% CI 19 300 to 37 700) fewer individuals. These estimates suggest that the annual saving to the government would be around £332 895 219 (95% CI £221 690 416 to £432 646 916) per year.

Indeed, increasing levels of youth NEET and rising social welfare benefit costs related to young people with mental health problems are key policy issues in the UK.^56^,^58^ Our analyses of the MCS population are pertinent to these debates, since MCS captures representative data from childhood on the UK’s young adults who are currently transitioning into the labour market. Our findings highlight the need for a life-course and social determinants prevention perspective. While the UK government has taken steps to expand early years support and has announced plans to address child poverty,^59^ more action is needed. Priorities should include improved access to parental mental health services, including perinatal and family-based care, anti-poverty strategies such as increasing child benefit, raising the minimum wage, strengthening universal credit protections, stronger support in education, particularly for adolescents at risk of exclusion.^60^ These should be coupled with guaranteed training places, apprenticeships and supported employment schemes for vulnerable youth.^61^ Without bold investment in upstream interventions, cycles of intergenerational disadvantage will continue to limit opportunities and may further undermine long-term economic stability.

## Conclusion

In conclusion, this study provides evidence that persistent exposure to childhood adversity, particularly household poverty and poor parental mental health, substantially increases the risk of being NEET on transition in early adulthood. These findings reinforce the importance of early family-centred interventions and the need for structural policies that support child development, educational attainment and the transition from school to work.

## Supplementary material

10.1136/bmjph-2025-003958 online supplemental appendix 1

## Data Availability

Data are available upon reasonable request.
